# A minimalist opportunist: the emergence of *Acinetobacter baumannii*

**DOI:** 10.1128/jb.00360-26

**Published:** 2026-09-29

**Authors:** Manon Janet-Maitre, Mario F. Feldman

**Affiliations:** 1 Department of Molecular Microbiology, Washington University School of Medicine in St. Louis 12275, St. Louis, Missouri, USA; Dartmouth College Geisel School of Medicine, Hanover, New Hampshire, USA

**Keywords:** opportunistic pathogen, antibiotic resistance, *Acinetobacter baumannii*, stress response

## Abstract

*Acinetobacter baumannii* has evolved from a relatively overlooked nosocomial pathogen into one of the most concerning multidrug-resistant threats worldwide. Its global dissemination, accelerated by healthcare-associated transmission and the emergence of carbapenem-resistant strains, has established *A. baumannii* as a critical priority pathogen. While initially studied primarily through the lens of antimicrobial resistance, growing evidence indicates that the remarkable success of *A. baumannii* cannot be attributed to resistance alone. Unlike many classical bacterial pathogens, *A. baumannii* lacks an extensive arsenal of virulence factors and major secretion systems. Instead, its success relies on a highly effective “persist and resist” strategy, combining intrinsic tolerance, extensive genomic plasticity, metabolic adaptability, and multilayered regulatory networks that enable survival under severe environmental and host-imposed stresses. This apparent simplicity, coupled with exceptional resilience, makes *A. baumannii* a particularly informative system for understanding how limited pathogenic determinants can generate profound clinical success. In this review, we highlight the key biological features that have established *A. baumannii* as a model system. We discuss milestone discoveries that have reshaped our understanding of its pathogenesis, emphasizing how this pathogen leverages adaptability rather than an extensive virulence arsenal to succeed as an opportunistic pathogen, expanding the field beyond antibiotic resistance toward broader questions of host-pathogen interaction and environmental adaptation. Finally, we outline major open questions in the field, including the contribution of intracellular reservoirs to chronic infection and treatment failure. Together, these perspectives position *A. baumannii* as a unique system for investigating how a seemingly minimalist pathogen achieves extraordinary adaptability and clinical success.

## INTRODUCTION

In the early 20th century, Martinus Beijerinck first described members of the *Acinetobacter* genus, which he named *Micrococcus calcoaceticus*. The name *Acinetobacter* (from the Greek *akinetos*, meaning “non-motile”) was proposed by Brisou and Prévot in 1954 and was later formally recognized by the Subcommittee on the Taxonomy of *Moraxella* and Allied Bacteria in 1971 (1). Although *Acinetobacter* spp. lack flagella and were initially classified as non-motile, it is now well established that they can exhibit forms of surface-associated motility (2). *Acinetobacter* species are ubiquitous in the environment; however, the opportunistic pathogen *A. baumannii* is predominantly associated with hospital settings.

As early as the 1970s, *A. baumannii* was recognized as a nosocomial pathogen due to its ability to adhere to plastic medical devices such as catheters and endotracheal tubes, as well as its capacity to withstand desiccation and persist on hospital surfaces (3). *A. baumannii* causes a wide range of infections in critically ill patients, including respiratory tract infections, particularly ventilator-associated pneumonia, as well as bloodstream, wound, and urinary tract infections (UTIs), often associated with catheter use (4, 5). These infections are especially prevalent in intensive care units (ICUs). Its rapid acquisition of resistance to multiple classes of antibiotics, including last-line carbapenems, has been documented since the 1980s, leading to the emergence of carbapenem-resistant *A. baumannii* (CRAB) (6, 7).

*A. baumannii* gained global attention during the Iraq War with the United States (from 2003 onward), where it earned the nickname “Iraqibacter”. Combat-related wounds and burns, combined with strained healthcare conditions, facilitated intra-hospital transmission and the spread of multidrug-resistant (MDR) strains (8–10). The repatriation of military and civilian casualties to the United Kingdom and the United States contributed to the global dissemination of these resistant strains, leading to outbreaks in hospitals worldwide (11–13).

Since the 2000s, *A. baumannii* has been recognized as one of the ESKAPEE pathogens (*Enterococcus faecium*, *Staphylococcus aureus*, *Klebsiella pneumoniae*, *Acinetobacter baumannii*, *Pseudomonas aeruginosa*, *Escherichia coli*, and *Enterobacter* spp.), a group of bacterial pathogens known for their ability to evade antimicrobial treatments. It is also classified as a “critical priority” pathogen by the World Health Organization due to the alarming rise in multidrug-resistant (MDR), extensively drug-resistant (XDR), and even pan-drug-resistant strains. In some countries, more than 80% of *A. baumannii* isolates exhibit resistance to commonly used antibiotics (14, 15).

More recently, the COVID-19 pandemic, caused by SARS-CoV-2, has further exacerbated the burden of *A. baumannii* infections. Severe COVID-19 cases often require intensive care, increasing the risk of secondary infections, particularly respiratory, bloodstream, and urinary tract infections (16, 17). Invasive mechanical ventilation significantly elevates the risk of ventilator-associated pneumonia, frequently due to secondary bacterial infections, with *A. baumannii* being among the most prevalent pathogens (17). Increased ICU occupancy, overwhelmed healthcare systems, and widespread antibiotic use during the pandemic contributed to outbreaks of MDR and CRAB strains (18, 19).

As a result, what was once considered an opportunistic hospital pathogen has evolved into a globally disseminated and highly resilient threat. With over 300,000 deaths attributable to *A. baumannii* in 2019, prolonged hospital stays averaging around 30 days (20, 21), high mortality rates, and limited therapeutic options, this pathogen represents a major burden on modern healthcare systems. There is an urgent need to better understand its biology and pathogenicity in order to develop effective alternative treatments.

## FEATURES THAT MAKE *A. BAUMANNII* A GREAT MODEL SYSTEM

The establishment of *A. baumannii* as a model system occurred alongside its rapid global spread, raising growing concern about antimicrobial resistance. This prompts a key question: why has *A. baumannii* developed multidrug resistance at such an exceptional pace?

Part of the answer lies in its intrinsic resistance. *A. baumannii* exhibits low outer membrane permeability, driven in part by the restrictive nature of outer membrane proteins such as OmpA (22–25), and expresses multiple efflux systems, some of which are constitutively active, that can actively expel a wide range of antibiotics (26–28).

**Fig 1:**
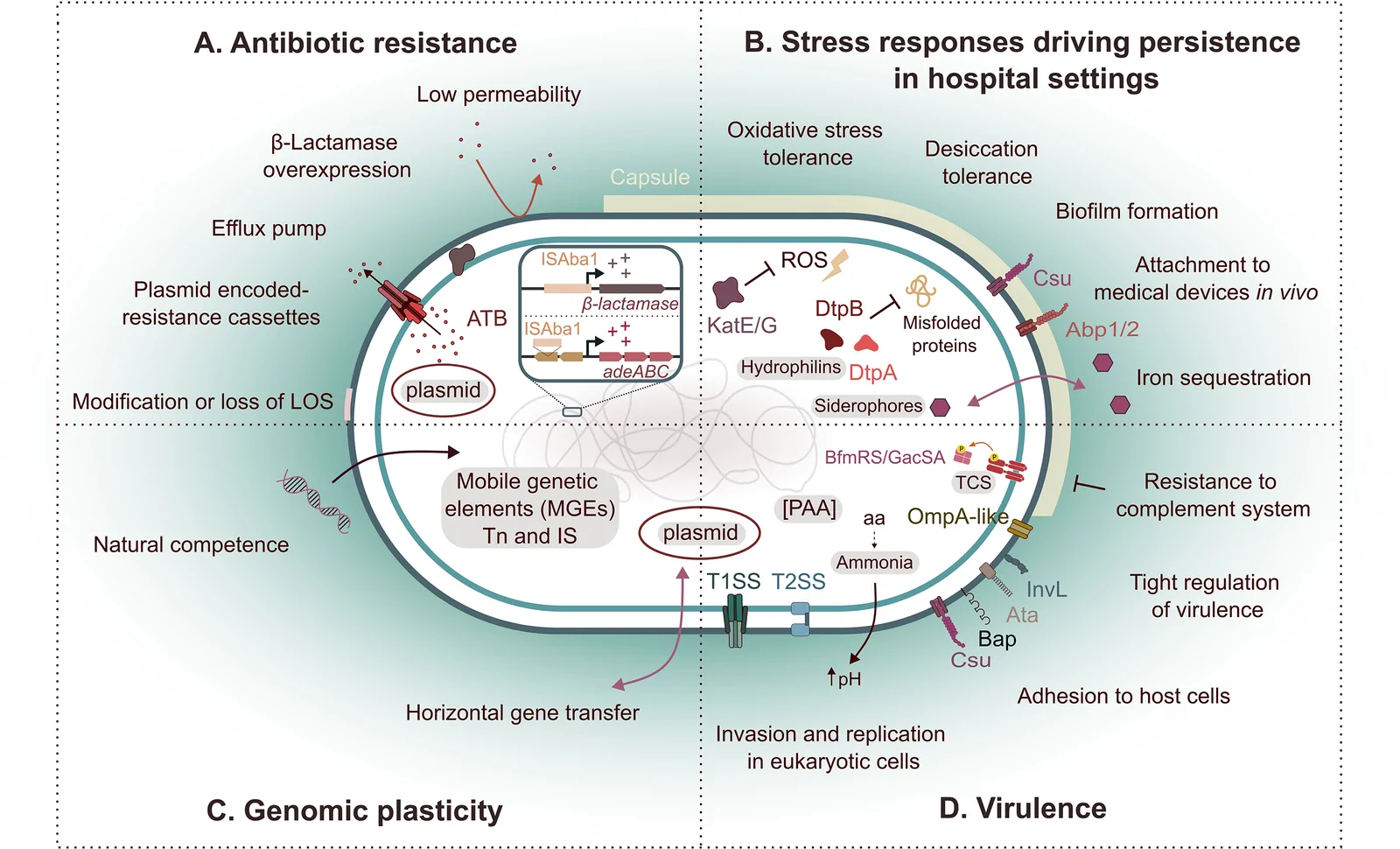
The toolkit of a successful opportunistic pathogen. *A. baumannii* relies on a limited yet highly integrated set of traits that collectively enable persistence in clinical environments and opportunistic infection. These include antibiotic resistance (**A**), stress responses involved in the success of *A. baumannii* in hospital settings (**B**), genomic plasticity (**C**), and host interaction and virulence strategies (**D**). aa, amino acids; ATB, antibiotics; IS, insertion sequences; LOS, lipooligosaccharide; PAA, phenylacetic acid; T1SS, type 1 secretion system; T2SS, type 2 secretion system; TCS, two-component systems; Tn, transposons.

Beyond these baseline defenses, its remarkable evolutionary success is driven by the combination of natural competence and high genomic plasticity (29). Mobile genetic elements (MGEs), including transposons and insertion sequences (ISs), play a central role in this process. While many *A. baumannii* isolates are not naturally competent, strains capable of natural transformation can acquire large fragments of exogenous DNA, potentially carrying multidrug-resistance determinants (30). These are then efficiently integrated, rearranged, and expressed within a highly recombinogenic genome, supported by mobile elements (29). Insertion sequences (ISs), a class of dynamic MGEs, play an important role in *A. baumannii* antibiotic resistance. For example, the insertion sequence ISAba1 has been shown to modulate *A. baumannii* antibiotic resistance in different ways, including its frequent insertion upstream of the chromosomal β-lactamase genes *bla*_OXA-51_, *bla*_OXA-23_, *bla*_ADC_, and *ampC* leading to their upregulation and subsequent increased resistance to carbapenems and cephalosporins (29, 31–34). Additionally, insertion of ISAba1 into the *adeS* gene, upstream of the *adeABC* operon, can lead to constitutive overexpression of the AdeABC efflux pump, thereby enhancing antibiotic resistance (Fig. 1) (35). Other genomic islands, including AbaR, can also modulate competence by inserting into *comM*, a critical gene in *A. baumannii competence*, further illustrating the complexity of these genomic interactions (36). Under antibiotic pressure, this combination of natural transformation and high genetic plasticity enables the rapid evolution of extensive, and sometimes unprecedented, resistance profiles. Additionally, the acquisition of plasmids harboring antibiotic resistance cassettes further drives *A. baumannii* genomic plasticity (37, 38).

In Gram-negative bacteria, resistance to colistin is commonly mediated by modifications of the lipid A component of lipopolysaccharide (LPS) (39). In *A. baumannii*, this typically involves the addition of a phosphoethanolamine (pEtN) moiety to the LPS by the phosphoethanolamine transferase EptA (PmrC), which reduces the negative charge of the bacterial outer membrane and decreases colistin binding (40). This modification is regulated by the two-component regulatory system (TCS) PmrAB, and clinical resistance to colistin is most commonly associated with mutations in *pmrAB* that result in constitutive activation of the system (41). *A. baumannii* can also employ similar LPS-modifying mechanisms. However, it additionally possesses a distinct strategy: it can completely lose its lipooligosaccharide (LOS), an LPS devoid of O-antigen, thereby effectively eliminating the target of colistin altogether (42, 43).

Understanding how these diverse mechanisms interact has been greatly facilitated by technological advances. In particular, next-generation sequencing has transformed *A. baumannii* research by enabling high-resolution phylogenetic analyses, tracking the spread of resistant lineages, and supporting the development of molecular tools. As a genetically tractable organism, it has become increasingly accessible for experimental studies.

These advances have, in turn, shifted the focus from resistance alone to a broader understanding of pathogenesis. Although individual virulence factors were described earlier, more systematic investigations into *A. baumannii* pathogenicity have only gained momentum in recent years. In parallel, the development of improved animal models has provided deeper insight into host-pathogen interactions and disease mechanisms.

Overall, although *A. baumannii* may not be a traditional model for understanding conserved processes and virulence strategies, its unique lifestyle and minimalist approach to pathogenesis, largely centered on its ability to withstand and adapt to diverse stresses, provide valuable insights into a distinct class of opportunistic pathogens. As a model system, studying *A. baumannii* pathogenesis challenges traditional concepts of virulence and may help broaden our understanding of opportunistic pathogens that rely on a limited repertoire of classical virulence factors yet remain highly successful pathogens.

## UNIQUE FEATURES AND MILESTONE DISCOVERIES

### Persistence in clinical settings

Since its emergence in clinical environments, *A. baumannii* has proven to be exceptionally well adapted to withstand and persist under hospital conditions. A defining feature of this pathogen is its ability to maintain viability under nutrient-deprived and desiccating conditions. While all strains exhibit substantial tolerance to desiccation, certain isolates can survive for several months in dry environments (44–46).

This phenotype is governed by a complex regulatory network, prominently involving the TCS BfmRS (47). Among its downstream effectors are the hydrophilins DtpA and DtpB, which play key roles in desiccation tolerance (46). Notably, recombinant DtpA has been shown to enhance desiccation resistance and protect enzymatic activity from inactivation. Additional factors contributing to this process include acylation of lipid A within LOS, which may increase membrane fluidity, as well as the presence of the capsular polysaccharide (48, 49).

Beyond environmental persistence, *A. baumannii* is a major contributor to medical device-associated infections, largely due to its ability to form robust biofilms on abiotic surfaces such as endotracheal tubes and stainless steel (50). These biofilms confer increased tolerance to environmental stresses (50, 51). Biofilm formation is driven primarily by the production of the extracellular matrix polymer poly-β-1,6-N-acetylglucosamine (PNAG) (52), in combination with surface-associated structures. Among these, Csu, a chaperone-usher pili (CUP), is critical for initial attachment and biofilm formation on abiotic surfaces (53–55). Additional CUP pili have been identified in many strains. These include Abp1 (or CUP1) and Abp2 (or CUP2) (56). Other surface appendages, including *Acinetobacter* trimeric autotransporter adhesin (Ata), have also been implicated in biofilm formation. Although their precise roles remain less clearly defined (Fig. 1) (57).

Importantly, most studies on biofilm formation have been conducted *in vitro*. However, *in vivo* models provide additional insight: insertion of medical tubing into the urinary and respiratory tracts leads to rapid coating with host proteins such as fibrin and fibrinogen (58–60). In this context, *A. baumannii* relies on specialized surface structures to colonize the device, including the invasin InvL, Ata, and the CUP pili Abp1 and Abp2, which mediate binding to fibrinogen-coated catheters and are essential for colonization during catheter-associated urinary tract infections (CAUTIs) (56, 61). Remarkably, the structural tip adhesin of the Abp1 pilus functions as a lectin, binding directly to the complex N-linked sugar moieties present on the fibrinogen molecule (56).

### *A. baumannii*: a paradigm of stress-adapted persistence

Rather than relying on a single dominant virulence determinant, this pathogen combines intrinsic tolerance with highly coordinated regulatory responses, enabling persistence across fluctuating and often hostile conditions. This versatility underpins its ability to thrive in both clinical and environmental reservoirs.

#### Oxidative and iron-limiting stress resilience

*A. baumannii* frequently encounters high levels of reactive oxygen species (ROS), arising from disinfectants such as hydrogen peroxide, desiccation, and host immune defenses during infection. Its notable tolerance to ROS relies on a robust arsenal of detoxifying enzymes, including catalases (KatG and KatE; Fig. 1) and superoxide dismutases, largely controlled by the global regulator OxyR (62, 63). More recently, the heat shock protein HslJ was shown to contribute to H_2_O_2_ tolerance in an OxyR-dependent manner (64). In addition, insertion of ISAba1 upstream of *katG* can drive ~20-fold overexpression, further enhancing hydrogen peroxide tolerance (65). Together, these mechanisms illustrate both regulatory and genetic routes to oxidative stress resilience.

Iron limitation represents another major challenge during infection. As transition metals such as iron are essential for virtually all forms of life, hosts restrict their availability through a process termed nutritional immunity to limit pathogen growth (66). *A. baumannii*, however, has evolved multiple strategies to overcome this constraint. Notably, it can produce up to ten distinct siderophores, specialized iron-chelating molecules (67). The best-characterized siderophore is the catechol-hydroxamate acinetobactin, which exists in two forms depending on environmental pH (68, 69) and is required for full virulence (70). The acinetobactin biosynthetic genes are highly conserved across *A. baumannii* strains. In addition, the bacterium produces other siderophores, including the conserved baumannoferrin and the rarer fimsbactins, although their precise roles in physiology and virulence remain less well defined (67, 71, 72). The importance of siderophore-mediated iron uptake has recently been exploited therapeutically, with the development of siderophore-antibiotic conjugates that hijack these uptake systems to restore antibacterial activity. A prominent example is cefiderocol, a siderophore cephalosporin novel antibiotic (73).

#### Capsule and phenotypic diversity

The capsule is a central virulence determinant in *A. baumannii*, conferring protection against desiccation, disinfectants, antibiotics, and host innate immunity, including resistance to serum complement (69, 74–77). Capsule biosynthesis is encoded by the highly variable K locus, with more than 200 identified types, underscoring extensive capsular diversity (78). Notably, the capsule also serves as a primary receptor for bacteriophages, contributing to restricted phage host range (79).

At the population level, *A. baumannii* exploits phase variation to generate phenotypic heterogeneity within isogenic populations. This bet-hedging strategy enables isogenic populations to diversify into subpopulations with distinct traits (80). In strain AB5075, two colony morphotypes, opaque and translucent, were described (48). These variants, which have also been observed in numerous other *A. baumannii* strains and clinical isolates but not in the domesticated strain ATCC 17978 (48, 81), differ markedly in capsule thickness, virulence, motility, biofilm formation, and antibiotic resistance. Opaque variants are hypervirulent, exhibiting increased capsule production and competitive fitness *in vivo* (81, 82). Switching frequency is influenced by cell density and regulated by the TetR-type regulator ABUW_1645 (82). This phenotype was also observed in other strains of *A. baumannii*. However, the molecular mechanisms and environmental triggers governing this switch remain incompletely understood.

Importantly, phase variation has practical implications for experimental design. Detection requires plating >200 CFU on low-agar media and visualization under specific lighting conditions (48). Given this inherent heterogeneity, experimental workflows should account for population diversity, either by quantifying switching frequencies or by inoculating cultures from multiple colonies to avoid biased phenotypic selection.

#### PAA: metabolism meets signaling

Beyond structural and phenotypic adaptations, *A. baumannii* integrates stress responses with metabolism and signaling networks. Canonical secondary messengers, including cAMP, c-di-GMP, and (p)ppGpp, contribute to global regulatory control (83–85). In parallel, the phenylacetic acid (PAA) degradation pathway has emerged as a key link between metabolism and stress adaptation. The *paa* operon is consistently identified as highly regulated across diverse conditions, including biofilm growth and environmental stress (86, 87), suggesting a central physiological role. Increasing evidence indicates that PAA functions beyond metabolism as a signaling molecule influencing biofilm formation, pili expression, antibiotic tolerance, and oxidative stress resistance, with measurable contributions to virulence (88). Functional studies further support this role, as disruption of *paa* genes impairs intracellular survival and desiccation tolerance by promoting entry into a viable but non-culturable (VBNC) state (89, 90). Despite these advances, the integration of PAA signaling within broader regulatory networks remains to be clarified.

#### Regulatory hubs

The versatility of *A. baumannii* ultimately relies on integrated regulatory systems that fine-tune stress responses. Among these, the TCSs BfmRS and GacSA act as central hubs. BfmRS controls a large regulon (>300 genes) and governs key traits including oxidative, osmotic, and heat stress responses, as well as capsule production, Csu pili assembly, and siderophore synthesis (91–94). These functions directly contribute to serum resistance and antibiotic tolerance. GacSA similarly regulates virulence-associated phenotypes such as biofilm formation and motility, and notably modulates expression of the *paa* operon (91, 95). In parallel, N-acyl homoserine lactone (AHL)-based quorum sensing further shapes virulence and community behaviors, although this system has been extensively reviewed elsewhere (96–98).

Collectively, *A. baumannii* persistence emerges from the interplay between intrinsic tolerance mechanisms, phenotypic diversification, metabolic flexibility, and multilayered regulatory control. These systems do not act in isolation but form an interconnected network that allows rapid adaptation to stress. Elucidating how these pathways are integrated and identifying points of vulnerability within this network remain a key challenges for understanding and ultimately controlling this highly resilient pathogen.

### Pathogenesis strategies of *A. baumannii*

In recent years, focus has shifted to the study of the pathogenicity and virulence of *A. baumannii*. This organism can be considered a “minimalist” pathogen: rather than relying on a large arsenal of canonical virulence factors, it leverages a limited set of determinants combined with versatility and highly efficient stress response mechanisms to survive and proliferate in host environments. Here, we do not aim to comprehensively review all described virulence factors, which have been extensively covered elsewhere (69, 91). Instead, we highlight key features that underpin the success of *A. baumannii* as a pathogen.

#### Adhesion to biotic surfaces

Adhesion to host cells represents a critical early step in *A. baumannii* pathogenesis. Individual strains express distinct repertoires of surface adhesins, enabling fine-tuned interactions with specific host tissues. Well-characterized factors involved in adhesion include the biofilm-associated protein Bap, the autotransporter Ata, and the outer membrane protein OmpA (99–101). More recently, the twin-arginine translocation (Tat) system has also been implicated in adhesion to both epithelial (A549) and endothelial (EA.hy926) cells (102). Additionally, InvL has been shown to mediate adhesion to kidney (MDCK) and bladder (5637/HTB-9) epithelial cells (Fig. 1) (61).

Other factors remain more controversial. Early studies suggested that the Csu CUP pili were required for attachment to abiotic surfaces but not to biotic ones (103). However, more recent work indicates a role for Csu pili in adhesion to epithelial cells and in lung colonization *in vivo* (104, 105). These discrepancies likely reflect strain-dependent differences and variable expression across conditions, highlighting the need for further investigation to clarify the role of Csu in host cell adhesion.

#### A facultative intracellular lifestyle

Increasing evidence now supports the view of *A. baumannii* as a facultative intracellular pathogen. Across multiple models, some strains and clinical isolates of *A. baumannii* have been shown to invade and replicate within diverse eukaryotic cells, including epithelial cells, endothelial cells, and macrophages (100, 106, 107) reviewed in reference 108. *In vivo*, *Acinetobacter*-containing vacuoles (ACVs) have been observed in infected mouse lungs. In macrophages, early resistance to acidic pH is critical for ACV establishment. Certain strains, such as Ab398, subsequently neutralize vacuolar acidification through the production of ammonia as a byproduct of amino acid catabolism (109). Indeed, *A. baumannii* cannot solely grow on sugars and mostly uses amino acids as a carbon source (110). The catabolism of amino acids results in the release of ammonia, with the subsequent alkalization of the extracellular milieu, which is essential for ACV formation in macrophages (109, 111, 112). Within ACVs, *A. baumannii* deploys additional strategies to counter host-imposed stresses, including activation of stress response pathways, metabolic reprogramming (e.g., PAA catabolism), shifts in carbon source utilization, and surface remodeling (89). For instance, overproduction of LpsB, an enzyme involved in LOS biosynthesis, decreases sensitivity to vancomycin, suggesting enhanced envelope integrity (89). The type I secretion system (T1SS) may also contribute to intracellular survival and replication (106). Transfer of bronchoalveolar lavage fluid containing infected host cells into naïve mice results in productive lung infection, indicating that intracellular *A. baumannii* remains viable and infectious. These findings suggest that intracellular reservoirs may contribute to persistence, immune evasion, and antibiotic tolerance, potentially leading to treatment failure (89). Interestingly, intracellular strategies vary by cell type. In endothelial cells, the invasive strain ABC141 does not neutralize vacuolar pH via ammonia production. Instead, intracellular replication depends on T1SS, while the type II secretion system (T2SS) is required for host cell invasion (113). In the urinary tract, *A. baumannii* can also form intracellular reservoirs within bladder epithelial cells (ABIRs), which persist after apparent infection clearance. Reinsertion of a sterile catheter can trigger recurrent infection seeded from these reservoirs (114). Although this facultative intracellular lifestyle has not been demonstrated across all *A. baumannii* strains (107, 109), its occurrence in diverse cellular and infection models suggests that it may have a significant impact on infection dynamics, persistence, and treatment outcomes.

#### Plasmid-chromosome crosstalk

Many *A. baumannii* strains harbor one or more plasmids, which are well known for carrying antibiotic resistance determinants. The rapid expansion of antimicrobial resistance is reflected in the evolution of the large conjugative plasmid pAB3, initially carrying a single resistance cassette in strain ATCC17978, into pAB04, which now contains multiple resistance determinants (37, 69).

Beyond resistance, plasmids play a broader role in regulating bacterial physiology, competitiveness, and virulence. For example, large conjugative plasmids (LCPs) have been shown to repress the type VI secretion system (T6SS), which in *A. baumannii* primarily mediates interbacterial competition (37). More recent studies further demonstrate that plasmids can influence both virulence and niche adaptation. In the clinical strain UPAB1, the presence of its LCP pAB5 enhances virulence in a catheter-associated urinary tract infection (CAUTI) model, whereas plasmid loss increases virulence in an acute pneumonia model (115). Plasmids also impact intracellular survival. In macrophages, the UPAB1 strain harboring pAB5 can replicate intracellularly, while its plasmid-cured counterpart is rapidly cleared (106). Another example of plasmid-chromosome crosstalk is the regulation of phase switching between the aforementioned virulent-opaque and avirulent-translucent states by the sRNA SrvS, which is encoded on the largest plasmid of AB5075, p1AB5075. SrvS not only regulates the switch frequency, but also which TetR-family transcriptional regulators mediate the switch.

Together, these findings highlight the intricate genetic and regulatory interplay between plasmids and the chromosome in shaping *A. baumannii* pathogenesis.

## CURRENT/FUTURE QUESTIONS

*A. baumannii* has evolved from an overlooked opportunistic pathogen into a globally significant multidrug-resistant threat. Yet, much of our current understanding still relies on a limited number of laboratory-adapted strains maintained for decades. While strains such as ATCC 19606 and ATCC 17978 have been instrumental, they do not capture the diversity of contemporary clinical isolates. The study of recent strains has already uncovered key traits, including phase variation and an intracellular lifestyle (48, 82, 106, 107), underscoring the need to systematically expand investigations beyond historical models.

Advancing our understanding of *A. baumannii* pathogenesis also relies on the development of relevant animal models. Widely used infection models, particularly acute murine sepsis and pneumonia (116, 117), have provided critical insights but incompletely reflect the chronic and often polymicrobial nature of *A. baumannii* infections. Alternative models, including wound, soft tissue, and catheter-associated urinary tract infection (CAUTI) systems (115, 118), as well as more recent chronic pneumonia models enabling sustained infection at clinically relevant inocula (119), offer improved frameworks to study host-pathogen interactions in chronic infections, antibiotic efficacy, and polymicrobial infections. This is particularly relevant given that polymicrobial infections may account for ~25% of *A. baumannii* cases (120).

A major conceptual advance in recent years is the recognition of *A. baumannii* as a facultative intracellular pathogen. Evidence from multiple models indicates that the bacterium can establish intracellular populations within host cells, including transient reservoirs in the lung and persistent reservoirs in bladder epithelial cells (ABIRs), capable of reseeding infection following catheter insertion (89, 114). These findings provide a plausible mechanistic basis for clinical observations, including prolonged hospital stays (∼30 days on average) and frequent treatment failure despite *in vitro* susceptibility (20, 21). However, the extent to which intracellular persistence contributes to human infection remains to be determined. This intracellular lifestyle should be considered when assessing treatment options and represents a key priority for future research.

These findings also raise questions about the origin of infecting strains and the role of host colonization. Specifically, the gastrointestinal tract has emerged as a major determinant of infection risk. Early work identified the gut as a reservoir (121), and more recent studies report colonization rates of up to 40% in hospitalized patients, with a high prevalence at ICU admission (122). Importantly, prior colonization is associated with a markedly increased risk of infection, up to 15-fold higher ([123, 124], reviewed in reference 125). Interestingly, age and feeding practices may also influence gut colonization, as healthy 4-month-old infants fed formula were reported to have a higher prevalence of *A. baumannii* than breastfed infants (126). Given the clear association between intestinal carriage and increased risk of *A. baumannii* infection, further investigation of the mechanisms underlying gut colonization and its contribution to disease is warranted. The recent development of a murine model to study *A. baumannii* gut colonization should provide an important tool for advancing this field (126). Together, these observations highlight the importance of colonization dynamics in both pathogenesis and transmission.

Given the limited therapeutic options available and in line with World Health Organization priorities, significant efforts are being made to develop alternative treatment strategies. Bacteriophage therapy has shown promising results against CRAB *in vitro*, in animal models, and in clinical case studies (127–131). Phage cocktails can broaden host range, particularly relevant given capsule diversity, and reduce resistance emergence, while also displaying anti-biofilm activity (79, 131). In addition, phage-antibiotic combinations can restore susceptibility to drugs such as ceftazidime and produce synergistic effects ([132–134], reviewed in reference 135).

Complementary approaches based on drug repurposing and antibiotic combinations offer more immediate translational potential. For instance, the non-steroidal anti-inflammatory drug (NSAID) diclofenac enhances *A. baumannii* susceptibility to colistin *in vitro* and *in vivo* (136–138), while various antibiotic combinations have demonstrated synergistic activity (139, 140). Although promising, these strategies require further validation in clinically relevant settings. Preventive strategies remain challenging, particularly due to the highly immunocompromised status of many affected patients. Nonetheless, passive immunization approaches have demonstrated protective effects in animal models, supporting the potential of antibody-based interventions (141–143).

Collectively, these findings reinforce a central paradigm: the success of *A. baumannii* is not driven by an extensive repertoire of classical virulence factors, but by its exceptional adaptability. Rather than relying on potent toxins or complex secretion systems, it deploys a versatile arsenal of stress response mechanisms that enable it to withstand environmental challenges, reprogram metabolism, and exploit diverse host niches, thereby supporting persistence across clinical settings. Understanding the basis of this adaptability will be essential for developing effective strategies to combat this resilient and minimalist pathogen.

## References

[1] Lessel EF. 1971. International Committee on Nomenclature of Bacteria Subcommittee on the Taxonomy of *Moraxella* and allied bacteria: Minutes of the Meeting, 11 August 1970. Room Constitution C, Maria-Isabel Hotel, Mexico City, Mexico. Int J Syst Bacteriol 21:213–214. doi:10.1099/00207713-21-2-213

[2] Eijkelkamp BA, Stroeher UH, Hassan KA, Papadimitrious MS, Paulsen IT, Brown MH, Lo R. 2011. Adherence and motility characteristics of clinical *Acinetobacter baumannii* isolates. FEMS Microbiol Lett 323:44–51. doi:10.1111/j.1574-6968.2011.02362.x 22092679

[3] Glew RH, Moellering RC, Kunz LJ. 1977. Infections with *Acinetobacter calcoaceticus* (*Herellea vaginicola*). Medicine (Baltimore) 56:79–98. doi:10.1097/00005792-197703000-00001 846390

[4] Buxton AE, Anderson RL, Werdegar D, Atlas E. 1978. Nosocomial respiratory tract infection and colonization with *Acinetobacter calcoaceticus*. Epidemiologic characteristics. Am J Med 65:507–513. doi:10.1016/0002-9343(78)90777-5 362918

[5] Bergogne-Bérézin E, Towner KJ. 1996. *Acinetobacter* spp. as nosocomial pathogens: microbiological, clinical, and epidemiological features. Clin Microbiol Rev 9:148–165. doi:10.1128/CMR.9.2.148 PMC172888 8964033

[6] Devaud M, Kayser FH, Bächi B. 1982. Transposon-mediated multiple antibiotic resistance in *Acinetobacter* strains. Antimicrob Agents Chemother 22:323–329. doi:10.1128/AAC.22.2.323 PMC183733 6100428

[7] Paton R, Miles RS, Hood J, Amyes SGB, Miles RS, Amyes SGB. 1993. ARI 1: beta-lactamase-mediated imipenem resistance in *Acinetobacter baumannii*. Int J Antimicrob Agents 2:81–87. doi:10.1016/0924-8579(93)90045-7 18611526

[8] Ronat J-B, Kakol J, Khoury MN, Berthelot M, Yun O, Brown V, Murphy RA. 2014. Highly drug-resistant pathogens implicated in burn-associated bacteremia in an Iraqi burn care unit. PLoS One 9:e101017. doi:10.1371/journal.pone.0101017 PMC4128596 25111170

[9] Sahli ZT, Bizri AR, Abu-sittah GS. 2016. Microbiology and risk factors associated with war-related wound infections in the Middle East. Epidemiol Infect 144:2848–2857. doi:10.1017/S0950268816000431 PMC9150393 26931769

[10] Abou Fayad A, Rizk A, El Sayed S, Kaddoura M, Jawad NK, Al-Attar A, Dewachi O, Nguyen VK, Sater ZA. 2023. Antimicrobial resistance and the Iraq wars: armed conflict as an underinvestigated pathway with growing significance. BMJ Glob Health 7:e010863. doi:10.1136/bmjgh-2022-010863 PMC9933488 36781284

[11] Scott P, Deye G, Srinivasan A, Murray C, Moran K, Hulten E, Fishbain J, Craft D, Riddell S, Lindler L, Mancuso J, Milstrey E, Bautista CT, Patel J, Ewell A, Hamilton T, Gaddy C, Tenney M, Christopher G, Petersen K, Endy T, Petruccelli B. 2007. An outbreak of multidrug-resistant *Acinetobacter baumannii*-calcoaceticus complex infection in the US military health care system associated with military operations in Iraq. Clin Infect Dis 44:1577–1584. doi:10.1086/518170 17516401

[12] Turton JF, Kaufmann ME, Gill MJ, Pike R, Scott PT, Fishbain J, Craft D, Deye G, Riddell S, Lindler LE, Pitt TL. 2006. Comparison of *Acinetobacter baumannii* isolates from the United Kingdom and the United States that were associated with repatriated casualties of the Iraq conflict. J Clin Microbiol 44:2630–2634. doi:10.1128/JCM.00547-06 PMC1489513 16825400

[13] O’Shea MK. 2012. *Acinetobacter* in modern warfare. Int J Antimicrob Agents 39:363–375. doi:10.1016/j.ijantimicag.2012.01.018 22459899

[14] Murray CJL, Ikuta KS, Sharara F, Swetschinski L, Robles Aguilar G, Gray A, Han C, Bisignano C, Rao P, Wool E, et al.. 2022. Global burden of bacterial antimicrobial resistance in 2019: a systematic analysis. The Lancet 399:629–655. doi:10.1016/S0140-6736(21)02724-0 PMC8841637 35065702

[15] WHO bacterial priority pathogens list. 2024. Bacterial pathogens of public health importance to guide research, development and strategies to prevent and control antimicrobial resistance. Available from: https://www.who.int/publications/i/item/9789240093461. Retrieved 22 Apr 2025. 10.1016/S1473-3099(25)00118-5 PMC12367593 40245910

[16] Zhang H, Zhang Y, Wu J, Li Y, Zhou X, Li X, Chen H, Guo M, Chen S, Sun F, Mao R, Qiu C, Zhu Z, Ai J, Zhang W. 2020. Risks and features of secondary infections in severe and critical ill COVID-19 patients. Emerging Microbes & Infections 9:1958–1964. doi:10.1080/22221751.2020.1812437 PMC8284966 32815458

[17] Gajic I, Jovicevic M, Popadic V, Trudic A, Kabic J, Kekic D, Ilic A, Klasnja S, Hadnadjev M, Popadic DJ, Andrijevic A, Prokic A, Tomasevic R, Ranin L, Todorovic Z, Zdravkovic M, Opavski N. 2023. The emergence of multi-drug-resistant bacteria causing healthcare-associated infections in COVID-19 patients: a retrospective multi-centre study. J Hosp Infect 137:1–7. doi:10.1016/j.jhin.2023.04.013 PMC10140260 37121488

[18] Gottesman T, Fedorowsky R, Yerushalmi R, Lellouche J, Nutman A. 2021. An outbreak of carbapenem-resistant *Acinetobacter baumannii* in a COVID-19 dedicated hospital. Infect Prev Pract 3:100113. doi:10.1016/j.infpip.2021.100113 PMC7794049 34316574

[19] O’Toole RF. 2021. The interface between COVID-19 and bacterial healthcare-associated infections. Clin Microbiol Infect 27:1772–1776. doi:10.1016/j.cmi.2021.06.001 PMC8182977 34111586

[20] Pogue JM, Zhou Y, Kanakamedala H, Cai B. 2022. Burden of illness in carbapenem-resistant *Acinetobacter baumannii* infections in US hospitals between 2014 and 2019. BMC Infect Dis 22:36. doi:10.1186/s12879-021-07024-4 PMC8740340 34991499

[21] Alotaibi T, Abuhaimed A, Alshahrani M, Albdelhady A, Almubarak Y, Almasari O. 2021. Prevalence of multidrug-resistant *Acinetobacter baumannii* in a critical care setting: a tertiary teaching hospital experience. SAGE Open Med 9:20503121211001144. doi:10.1177/20503121211001144 PMC7968016 33796296

[22] Kwon HI, Kim S, Oh MH, Na SH, Kim YJ, Jeon YH, Lee JC. 2017. Outer membrane protein A contributes to antimicrobial resistance of *Acinetobacter baumannii* through the OmpA-like domain. J Antimicrob Chemother 72:3012–3015. doi:10.1093/jac/dkx257 28981866

[23] Sugawara E, Nikaido H. 2012. OmpA is the principal nonspecific slow porin of *Acinetobacter baumannii*. J Bacteriol 194:4089–4096. doi:10.1128/JB.00435-12 PMC3416538 22636785

[24] Iyer R, Moussa SH, Durand-Réville TF, Tommasi R, Miller A. 2018. *Acinetobacter baumannii* OmpA is a selective antibiotic permeant porin. ACS Infect Dis 4:373–381. doi:10.1021/acsinfecdis.7b00168 29260856

[25] Scribano D, Cheri E, Pompilio A, Di Bonaventura G, Belli M, Cristina M, Sansone L, Zagaglia C, Sarshar M, Palamara AT, Ambrosi C. 2024. *Acinetobacter baumannii* OmpA-like porins: functional characterization of bacterial physiology, antibiotic-resistance, and virulence. Commun Biol 7:948. doi:10.1038/s42003-024-06645-0 PMC11303520 39107399

[26] Damier-Piolle L, Magnet S, Brémont S, Lambert T, Courvalin P. 2008. AdeIJK, a resistance-nodulation-cell division pump effluxing multiple antibiotics in *Acinetobacter baumannii*. Antimicrob Agents Chemother 52:557–562. doi:10.1128/AAC.00732-07 PMC2224764 18086852

[27] Magnet S, Courvalin P, Lambert T. 2001. Resistance-nodulation-cell division-type efflux pump involved in aminoglycoside resistance in *Acinetobacter baumannii* strain BM4454. Antimicrob Agents Chemother 45:3375–3380. doi:10.1128/AAC.45.12.3375-3380.2001 PMC90840 11709311

[28] Naidu V, Bartczak A, Brzoska AJ, Lewis P, Eijkelkamp BA, Paulsen IT, Elbourne LDH, Hassan KA. 2023. Evolution of RND efflux pumps in the development of a successful pathogen. Drug Resist Updat 66:100911. doi:10.1016/j.drup.2022.100911 36592567

[29] Cain AK, Hamidian M. 2023. Portrait of a killer: Uncovering resistance mechanisms and global spread of *Acinetobacter baumannii**.* PLoS Pathog 19:e1011520. doi:10.1371/journal.ppat.1011520 PMC10414682 37561719

[30] Hu Y, He L, Tao X, Meng F, Zhang J. 2019. High DNA uptake capacity of international clone II *Acinetobacter baumannii* detected by a novel planktonic natural transformation assay. Front Microbiol 10:2165. doi:10.3389/fmicb.2019.02165 PMC6768954 31616393

[31] Hamidian M, Hall RM. 2013. ISAba1 targets a specific position upstream of the intrinsic ampC gene of *Acinetobacter baumannii* leading to cephalosporin resistance. J Antimicrob Chemother 68:2682–2683. doi:10.1093/jac/dkt233 23788477

[32] Raible KM, Sen B, Law N, Bias TE, Emery CL, Ehrlich GD, Joshi SG. 2017. Molecular characterization of β-lactamase genes in clinical isolates of carbapenem-resistant *Acinetobacter baumannii*. Ann Clin Microbiol Antimicrob 16:75. doi:10.1186/s12941-017-0248-3 PMC5691885 29145853

[33] Saranathan R, Kumari R, Kalaivani R, Suresh S, Rani A, Purty S, Prashanth K. 2017. Detection of ISAba1 in association with a novel allelic variant of the β-lactamase ADC-82 and class D β-lactamase genes mediating carbapenem resistance among the clinical isolates of MDR *A. baumannii*. J Med Microbiol 66:103–111. doi:10.1099/jmm.0.000395 28260590

[34] Al-Agamy MH, Khalaf NG, Tawfick MM, Shibl AM, El Kholy A. 2014. Molecular characterization of carbapenem-insensitive *Acinetobacter baumannii* in Egypt. Int J Infect Dis 22:49–54. doi:10.1016/j.ijid.2013.12.004 24607428

[35] Jo J, Ko KS. 2021. Tigecycline heteroresistance and resistance mechanism in clinical isolates of *Acinetobacter baumannii*. Microbiol Spectr 9:e0101021. doi:10.1128/Spectrum.01010-21 PMC8557860 34523993

[36] Tuffet R, Carvalho G, Godeux A-S, Mazzamurro F, Rocha EPC, Laaberki M-H, Venner S, Charpentier X. 2024. Manipulation of natural transformation by AbaR-type islands promotes fixation of antibiotic resistance in *Acinetobacter baumannii*. Proc Natl Acad Sci USA 121:e2409843121. doi:10.1073/pnas.2409843121 PMC11441513 39288183

[37] Weber BS, Ly PM, Irwin JN, Pukatzki S, Feldman MF. 2015. A multidrug resistance plasmid contains the molecular switch for type VI secretion in *Acinetobacter baumannii**.* Proc Natl Acad Sci USA 112:9442–9447. doi:10.1073/pnas.1502966112 PMC4522760 26170289

[38] Leungtongkam U, Thummeepak R, Tasanapak K, Sitthisak S. 2018. Acquisition and transfer of antibiotic resistance genes in association with conjugative plasmid or class 1 integrons of *Acinetobacter baumannii**.* PLoS One 13:e0208468. doi:10.1371/journal.pone.0208468 PMC6283642 30521623

[39] Islam MM, Jung DE, Shin WS, Oh MH. 2024. Colistin resistance mechanism and management strategies of colistin-resistant *Acinetobacter baumannii* infections. Pathogens 13:1049. doi:10.3390/pathogens13121049 PMC11728550 39770308

[40] Novović K, Jovčić B. 2023. Colistin resistance in *Acinetobacter baumannii:* molecular mechanisms and epidemiology. Antibiotics (Basel) 12:516. doi:10.3390/antibiotics12030516 PMC10044110 36978383

[41] Beceiro A, Llobet E, Aranda J, Bengoechea JA, Doumith M, Hornsey M, Dhanji H, Chart H, Bou G, Livermore DM, Woodford N. 2011. Phosphoethanolamine modification of lipid A in colistin-resistant variants of *Acinetobacter baumannii* mediated by the pmrAB two-component regulatory system. Antimicrob Agents Chemother 55:3370–3379. doi:10.1128/AAC.00079-11 PMC3122444 21576434

[42] Moffatt JH, Harper M, Harrison P, Hale JDF, Vinogradov E, Seemann T, Henry R, Crane B, St. Michael F, Cox AD, Adler B, Nation RL, Li J, Boyce JD. 2010. Colistin resistance in *Acinetobacter baumannii* is mediated by complete loss of lipopolysaccharide production. Antimicrob Agents Chemother 54:4971–4977. doi:10.1128/AAC.00834-10 PMC2981238 20855724

[43] Boll JM, Crofts AA, Peters K, Cattoir V, Vollmer W, Davies BW, Trent MS. 2016. A penicillin-binding protein inhibits selection of colistin-resistant, lipooligosaccharide-deficient *Acinetobacter baumannii*. Proc Natl Acad Sci USA 113:E6228–E6237. doi:10.1073/pnas.1611594113 PMC5068286 27681618

[44] Wendt C, Dietze B, Dietz E, Rüden H. 1997. Survival of *Acinetobacter baumannii* on dry surfaces. J Clin Microbiol 35:1394–1397. doi:10.1128/jcm.35.6.1394-1397.1997 PMC229756 9163451

[45] Jawad A, Seifert H, Snelling AM, Heritage J, Hawkey PM. 1998. Survival of *Acinetobacter baumannii* on dry surfaces: comparison of outbreak and sporadic isolates. J Clin Microbiol 36:1938–1941. doi:10.1128/JCM.36.7.1938-1941.1998 PMC104956 9650940

[46] Green ER, Fakhoury JN, Monteith AJ, Pi H, Giedroc DP, Skaar EP. 2022. Bacterial hydrophilins promote pathogen desiccation tolerance. Cell Host Microbe 30:975–987. doi:10.1016/j.chom.2022.03.019 PMC9283220 35413266

[47] Farrow JM, Wells G, Pesci EC. 2018. Desiccation tolerance in *Acinetobacter baumannii* is mediated by the two-component response regulator BfmR. PLoS One 13:e0205638. doi:10.1371/journal.pone.0205638 PMC6181384 30308034

[48] Tipton KA, Dimitrova D, Rather PN. 2015. Phase-variable control of multiple phenotypes in *Acinetobacter baumannii* strain AB5075. J Bacteriol 197:2593–2599. doi:10.1128/JB.00188-15 PMC4518826 26013481

[49] Boll JM, Tucker AT, Klein DR, Beltran AM, Brodbelt JS, Davies BW, Trent MS. 2015. Reinforcing lipid A acylation on the cell surface of *Acinetobacter baumannii* promotes cationic antimicrobial peptide resistance and desiccation survival. mBio 6:e00478–15. doi:10.1128/mBio.00478-15 PMC4442142 25991684

[50] Greene C, Wu J, Rickard AH, Xi C. 2016. Evaluation of the ability of *Acinetobacter baumannii* to form biofilms on six different biomedical relevant surfaces. Lett Appl Microbiol 63:233–239. doi:10.1111/lam.12627 PMC7057210 27479925

[51] Greene C, Vadlamudi G, Newton D, Foxman B, Xi C. 2016. The influence of biofilm formation and multidrug resistance on environmental survival of clinical and environmental isolates of *Acinetobacter baumannii*. Am J Infect Control 44:e65–71. doi:10.1016/j.ajic.2015.12.012 PMC6993530 26851196

[52] Choi AHK, Slamti L, Avci FY, Pier GB, Maira-Litrán T. 2009. The pgaABCD locus of *Acinetobacter baumannii* encodes the production of poly-beta-1-6-N-acetylglucosamine, which is critical for biofilm formation. J Bacteriol 191:5953–5963. doi:10.1128/JB.00647-09 PMC2747904 19633088

[53] Tomaras AP, Dorsey CW, Edelmann RE, Actis LA. 2003. Attachment to and biofilm formation on abiotic surfaces by *Acinetobacter baumannii*: involvement of a novel chaperone-usher pili assembly system. Microbiology (Reading) 149:3473–3484. doi:10.1099/mic.0.26541-0 14663080

[54] Pakharukova N, Tuittila M, Paavilainen S, Malmi H, Parilova O, Teneberg S, Knight SD, Zavialov AV. 2018. Structural basis for *Acinetobacter baumannii* biofilm formation. Proc Natl Acad Sci USA 115:5558–5563. doi:10.1073/pnas.1800961115 PMC6003481 29735695

[55] Malmi H, Pakharukova N, Paul B, Tuittila M, Ahmad I, Knight SD, Uhlin BE, Ghosal D, Zavialov AV. 2026. Antiparallel stacking of Csu pili drives *Acinetobacter baumannii* 3D biofilm assembly. Nat Commun 17:2508. doi:10.1038/s41467-026-68860-z PMC12996565 41654547

[56] Tamadonfar KO, Di Venanzio G, Pinkner JS, Dodson KW, Kalas V, Zimmerman MI, Bazan Villicana J, Bowman GR, Feldman MF, Hultgren SJ. 2023. Structure-function correlates of fibrinogen binding by *Acinetobacter* adhesins critical in catheter-associated urinary tract infections. Proc Natl Acad Sci USA 120:e2212694120. doi:10.1073/pnas.2212694120 PMC9942807 36652481

[57] Bentancor LV, Camacho-Peiro A, Bozkurt-Guzel C, Pier GB, Maira-Litrán T. 2012. Identification of Ata, a multifunctional trimeric autotransporter of *Acinetobacter baumannii*. J Bacteriol 194:3950–3960. doi:10.1128/JB.06769-11 PMC3416510 22609912

[58] Harbison J, Collins D, Lynch V, McNicholas WT. 1999. Acute stridor due to an upper tracheal membrane following endotracheal intubation. Eur Respir J 14:1238–1238. doi:10.1183/09031936.99.14512389 10596719

[59] Andersen MJ, Fong C, La Bella AA, Molina JJ, Molesan A, Champion MM, Howell C, Flores-Mireles AL. 2022. Inhibiting host-protein deposition on urinary catheters reduces associated urinary tract infections. eLife 11:e75798. doi:10.7554/eLife.75798 PMC8986317 35348114

[60] Flores-Mireles AL, Walker JN, Bauman TM, Potretzke AM, Schreiber HL, Park AM, Pinkner JS, Caparon MG, Hultgren SJ, Desai A. 2016. Fibrinogen release and deposition on urinary catheters placed during urological procedures. J Urol 196:416–421. doi:10.1016/j.juro.2016.01.100 PMC4965327 26827873

[61] Jackson-Litteken CD, Di Venanzio G, Le N-H, Scott NE, Djahanschiri B, Distel JS, Pardue EJ, Ebersberger I, Feldman MF. 2022. InvL, an invasin-like adhesin, is a type II secretion system substrate required for *Acinetobacter baumannii* uropathogenesis. mBio 13:e00258–22. doi:10.1128/mbio.00258-22 PMC9245377 35638734

[62] Sun D, Crowell SA, Harding CM, De Silva PM, Harrison A, Fernando DM, Mason KM, Santana E, Loewen PC, Kumar A, Liu Y. 2016. KatG and KatE confer *Acinetobacter* resistance to hydrogen peroxide but sensitize bacteria to killing by phagocytic respiratory burst. Life Sci 148:31–40. doi:10.1016/j.lfs.2016.02.015 PMC4792659 26860891

[63] Juttukonda LJ, Green ER, Lonergan ZR, Heffern MC, Chang CJ, Skaar EP. 2019. *Acinetobacter baumannii* OxyR regulates the transcriptional response to hydrogen peroxide. Infect Immun 87:e00413-18. doi:10.1128/IAI.00413-18 PMC6300632 30297527

[64] Scribano D, Pasqua M, Limongi D, Nencioni L, Palamara AT, Ambrosi C. 2025. The periplasmic protein HslJ is the first-line of defense against oxidative stress in *Acinetobacter baumannii*. Biol Res 58:2. doi:10.1186/s40659-025-00584-8 PMC11724541 39794876

[65] Wright MS, Mountain S, Beeri K, Adams MD. 2017. Assessment of insertion sequence mobilization as an adaptive response to oxidative stress in *Acinetobacter baumannii* using IS-seq. J Bacteriol 199:00833–16. doi:10.1128/JB.00833-16 PMC5388817 28193905

[66] Murdoch CC, Skaar EP. 2022. Nutritional immunity: the battle for nutrient metals at the host-pathogen interface. Nat Rev Microbiol 20:657–670. doi:10.1038/s41579-022-00745-6 PMC9153222 35641670

[67] Sheldon JR, Skaar EP. 2020. *Acinetobacter baumannii* can use multiple siderophores for iron acquisition, but only acinetobactin is required for virulence. PLoS Pathog 16:e1008995. doi:10.1371/journal.ppat.1008995 PMC7595644 33075115

[68] Shapiro JA, Wencewicz TA. 2016. Acinetobactin isomerization enables adaptive iron acquisition in *Acinetobacter baumannii* through pH-triggered siderophore swapping. ACS Infect Dis 2:157–168. doi:10.1021/acsinfecdis.5b00145 27624967

[69] Harding CM, Hennon SW, Feldman MF. 2018. Uncovering the mechanisms of *Acinetobacter baumannii* virulence. Nat Rev Microbiol 16:91–102. doi:10.1038/nrmicro.2017.148 PMC6571207 29249812

[70] Gaddy JA, Arivett BA, McConnell MJ, López-Rojas R, Pachón J, Actis LA. 2012. Role of acinetobactin-mediated iron acquisition functions in the interaction of *Acinetobacter baumannii* strain ATCC 19606T with human lung epithelial cells, *Galleria mellonella* caterpillars, and mice. Infect Immun 80:1015–1024. doi:10.1128/IAI.06279-11 PMC3294665 22232188

[71] Antunes LCS, Imperi F, Towner KJ, Visca P. 2011. Genome-assisted identification of putative iron-utilization genes in *Acinetobacter baumannii* and their distribution among a genotypically diverse collection of clinical isolates. Res Microbiol 162:279–284. doi:10.1016/j.resmic.2010.10.010 21144895

[72] Zhang R, Li D, Fang H, Xie Q, Tang H, Chen L. 2025. Iron-dependent mechanisms in *Acinetobacter baumannii*: pathogenicity and resistance. JAC Antimicrob Resist 7:dlaf039. doi:10.1093/jacamr/dlaf039 PMC11920509 40110557

[73] Ito A, Sato T, Ota M, Takemura M, Nishikawa T, Toba S, Kohira N, Miyagawa S, Ishibashi N, Matsumoto S, Nakamura R, Tsuji M, Yamano Y. 2018. *In vitro* antibacterial properties of cefiderocol, a novel siderophore cephalosporin, against gram-negative bacteria. Antimicrob Agents Chemother 62:e01454-17. doi:10.1128/AAC.01454-17 PMC5740388 29061741

[74] Iwashkiw JA, Seper A, Weber BS, Scott NE, Vinogradov E, Stratilo C, Reiz B, Cordwell SJ, Whittal R, Schild S, Feldman MF. 2012. Identification of a general O-linked protein glycosylation system in *Acinetobacter baumannii* and its role in virulence and biofilm formation. PLoS Pathog 8:e1002758. doi:10.1371/journal.ppat.1002758 PMC3369928 22685409

[75] Lees-Miller RG, Iwashkiw JA, Scott NE, Seper A, Vinogradov E, Schild S, Feldman MF. 2013. A common pathway for O-linked protein-glycosylation and synthesis of capsule in *Acinetobacter baumannii*. Mol Microbiol 89:816–830. doi:10.1111/mmi.12300 23782391

[76] Singh JK, Adams FG, Brown MH. 2019. Diversity and function of capsular polysaccharide in *Acinetobacter baumannii*. Front Microbiol 9. doi:10.3389/fmicb.2018.03301 PMC6333632 30687280

[77] Geisinger E, Isberg RR. 2015. Antibiotic modulation of capsular exopolysaccharide and virulence in *Acinetobacter baumannii*. PLoS Pathog 11:e1004691. doi:10.1371/journal.ppat.1004691 PMC4334535 25679516

[78] Cahill SM, Hall RM, Kenyon JJ. 2022. An update to the database for *Acinetobacter baumannii* capsular polysaccharide locus typing extends the extensive and diverse repertoire of genes found at and outside the K locus. Microb Genom 8:000878. doi:10.1099/mgen.0.000878 PMC9676051 36214673

[79] Blasco L, Bleriot I, González de Aledo M, Fernández-García L, Pacios O, Oliveira H, López M, Ortiz-Cartagena C, Fernández-Cuenca F, Pascual Á, Martínez-Martínez L, Pachón J, Azeredo J, Tomás M. 2022. Development of an anti-*Acinetobacter baumannii* biofilm phage cocktail: genomic adaptation to the host. Antimicrob Agents Chemother 66:e01923–21. doi:10.1128/aac.01923-21 PMC8923231 35041503

[80] Reyes Ruiz LM, Williams CL, Tamayo R. 2020. Enhancing bacterial survival through phenotypic heterogeneity. PLoS Pathog 16:e1008439. doi:10.1371/journal.ppat.1008439 PMC7241687 32437427

[81] Ahmad I, Karah N, Nadeem A, Wai SN, Uhlin BE. 2019. Analysis of colony phase variation switch in *Acinetobacter baumannii* clinical isolates. PLoS One 14:e0210082. doi:10.1371/journal.pone.0210082 PMC6319719 30608966

[82] Chin CY, Tipton KA, Farokhyfar M, Burd EM, Weiss DS, Rather PN. 2018. A high-frequency phenotypic switch links bacterial virulence and environmental survival in *Acinetobacter baumannii*. Nat Microbiol 3:563–569. doi:10.1038/s41564-018-0151-5 PMC5921939 29693659

[83] de Dios R, Forsyth VS, Harkova LG, Schache KJ, Hsueh BY, Strat H, Riley B, Rubio A, Pérez-Pulido AJ, Waters CM, Mobley HLT, Crépin S, McCarthy RR. 2026. Network-level divergence in cyclic di-GMP signalling drives ecological versatility in *Acinetobacter baumannii**.* NPJ Biofilms Microbiomes 12:112. doi:10.1038/s41522-026-00968-y PMC13243610 41957030

[84] Cui B, Peng G, Wang L-E, Deng Y. 2025. Signaling in *Acinetobacter baumannii*: quorum sensing and nucleotide second messengers. Comput Struct Biotechnol J 27:2168–2175. doi:10.1016/j.csbj.2025.05.032 PMC12162036 40510767

[85] Harkova LG, de Dios R, Rubio-Valle A, Pérez-Pulido AJ, McCarthy RR. 2024. Cyclic AMP is a global virulence regulator governing inter and intrabacterial signalling in *Acinetobacter baumannii*. PLoS Pathog 20:e1012529. doi:10.1371/journal.ppat.1012529 PMC11410210 39241032

[86] Rumbo-Feal S, Gómez MJ, Gayoso C, Álvarez-Fraga L, Cabral MP, Aransay AM, Rodríguez-Ezpeleta N, Fullaondo A, Valle J, Tomás M, Bou G, Poza M. 2013. Whole transcriptome analysis of *Acinetobacter baumannii* assessed by RNA-sequencing reveals different mRNA expression profiles in biofilm compared to planktonic cells. PLoS One 8:e72968. doi:10.1371/journal.pone.0072968 PMC3758355 24023660

[87] Müller GL, Tuttobene M, Altilio M, Martínez Amezaga M, Nguyen M, Cribb P, Cybulski LE, Ramírez MS, Altabe S, Mussi MA. 2017. Light modulates metabolic pathways and other novel physiological traits in the human pathogen *Acinetobacter baumannii**.* J Bacteriol 199. doi:10.1128/JB.00011-17 PMC5405214 28289081

[88] Hooppaw AJ, McGuffey JC, Di Venanzio G, Ortiz-Marquez JC, Weber BS, Lightly TJ, van Opijnen T, Scott NE, Cardona ST, Feldman MF. 2022. The phenylacetic acid catabolic pathway regulates antibiotic and oxidative stress responses in *Acinetobacter*. mBio 13. doi:10.1128/mbio.01863-21 PMC9239106 35467424

[89] Janet-Maitre M, Di Venanzio G, Jackson-Litteken CD, Scott NE, Feldman MF. 2025. Intracellular *Acinetobacter baumannii* acts as a transient reservoir in lung infection via a “persist and resist” strategy. Proc Natl Acad Sci USA 122:e2511369122. doi:10.1073/pnas.2511369122 PMC12718349 41364768

[90] Harkova LG, de Dios R, McCarthy RR. 2026. Phenylacetic acid mediates *Acinetobacter baumannii* entry into a viable but non-culturable state. Microbiology 172:001650. doi:10.1099/mic.0.001650 PMC12811014 41542904

[91] Morris FC, Dexter C, Kostoulias X, Uddin MI, Peleg AY. 2019. The mechanisms of disease caused by *Acinetobacter baumannii**.* Front Microbiol 10. doi:10.3389/fmicb.2019.01601 PMC6650576 31379771

[92] Mendes SG, Combo SI, Allain T, Domingues S, Buret AG, Da Silva GJ. 2023. Co-regulation of biofilm formation and antimicrobial resistance in *Acinetobacter baumannii*: from mechanisms to therapeutic strategies. Eur J Clin Microbiol Infect Dis 42:1405–1423. doi:10.1007/s10096-023-04677-8 PMC10651561 37897520

[93] Palethorpe S, Farrow JM, Wells G, Milton ME, Actis LA, Cavanagh J, Pesci EC. 2022. *Acinetobacter baumannii* regulates its stress responses via the BfmRS two-component regulatory system. J Bacteriol 204:e0049421. doi:10.1128/JB.00494-21 PMC8846317 34871031

[94] Geisinger E, Mortman NJ, Vargas-Cuebas G, Tai AK, Isberg RR. 2018. A global regulatory system links virulence and antibiotic resistance to envelope homeostasis in *Acinetobacter baumannii**.* PLoS Pathog 14:e1007030. doi:10.1371/journal.ppat.1007030 PMC5967708 29795704

[95] Cerqueira GM, Kostoulias X, Khoo C, Aibinu I, Qu Y, Traven A, Peleg AY. 2014. A global virulence regulator in *Acinetobacter baumannii* and its control of the phenylacetic acid catabolic pathway. J Infect Dis 210:46–55. doi:10.1093/infdis/jiu024 24431277

[96] Sun X, Ni Z, Tang J, Ding Y, Wang X, Li F. 2021. The abaI/abaR quorum sensing system effects on pathogenicity in *Acinetobacter baumannii*. Front Microbiol 12. doi:10.3389/fmicb.2021.679241 PMC8312687 34322102

[97] Zhong S, He S. 2021. Quorum sensing inhibition or quenching in *Acinetobacter baumannii:* the novel therapeutic strategies for new drug development. Front Microbiol 12. doi:10.3389/fmicb.2021.558003 PMC7882596 33597937

[98] López M, Blasco L, Gato E, Perez A, Fernández-Garcia L, Martínez-Martinez L, Fernández-Cuenca F, Rodríguez-Baño J, Pascual A, Bou G, Tomás M. 2017. Response to bile salts in clinical strains of *Acinetobacter baumannii* lacking the AdeABC efflux pump: virulence associated with quorum sensing. Front Cell Infect Microbiol 7:143. doi:10.3389/fcimb.2017.00143 PMC5423435 28536672

[99] Brossard KA, Campagnari AA. 2012. The *Acinetobacter baumannii* biofilm-associated protein plays a role in adherence to human epithelial cells. Infect Immun 80:228–233. doi:10.1128/IAI.05913-11 PMC3255684 22083703

[100] Choi CH, Lee JS, Lee YC, Park TI, Lee JC. 2008. *Acinetobacter baumannii* invades epithelial cells and outer membrane protein A mediates interactions with epithelial cells. BMC Microbiol 8:216. doi:10.1186/1471-2180-8-216 PMC2615016 19068136

[101] Tram G, Poole J, Adams FG, Jennings MP, Eijkelkamp BA, Atack JM. 2021. The *Acinetobacter baumannii* autotransporter adhesin ata recognizes host glycans as high-affinity receptors. ACS Infect Dis 7:2352–2361. doi:10.1021/acsinfecdis.1c00021 34339169

[102] Debruyne C, Hodge L, Hokamp K, Kröger C, Ershova AS, Salcedo SP. 2025. Invasive *Acinetobacter baumannii* ABC141 strain relies on the twin-arginine translocation export system for adhesion to host cells. Microbiology 171:001630. doi:10.1099/mic.0.001630 PMC12687288 41363066

[103] de Breij A, Gaddy J, van der Meer J, Koning R, Koster A, van den Broek P, Actis L, Nibbering P, Dijkshoorn L. 2009. CsuA/BABCDE-dependent pili are not involved in the adherence of *Acinetobacter baumannii* ATCC19606(T) to human airway epithelial cells and their inflammatory response. Res Microbiol 160:213–218. doi:10.1016/j.resmic.2009.01.002 19530313

[104] Ahmad I, Nadeem A, Mushtaq F, Zlatkov N, Shahzad M, Zavialov AV, Wai SN, Uhlin BE. 2023. Csu pili dependent biofilm formation and virulence of *Acinetobacter baumannii*. NPJ Biofilms Microbiomes 9:101. doi:10.1038/s41522-023-00465-6 PMC10721868 38097635

[105] Chen C-L, Dudek A, Liang Y-H, Janapatla RP, Lee H-Y, Hsu L, Kuo H-Y, Chiu C-H. 2022. D-mannose-sensitive pilus of *Acinetobacter baumannii* is linked to biofilm formation and adherence onto respiratory tract epithelial cells. J Microbiol Immunol Infect 55:69–79. doi:10.1016/j.jmii.2021.01.008 33610507

[106] Sycz G, Di Venanzio G, Distel JS, Sartorio MG, Le N-H, Scott NE, Beatty WL, Feldman MF. 2021. Modern *Acinetobacter baumannii* clinical isolates replicate inside spacious vacuoles and egress from macrophages. PLoS Pathog 17:e1009802. doi:10.1371/journal.ppat.1009802 PMC8376066 34370792

[107] Rubio T, Gagné S, Debruyne C, Dias C, Cluzel C, Mongellaz D, Rousselle P, Göttig S, Seifert H, Higgins PG, Salcedo SP. 2022. Incidence of an intracellular multiplication niche among *Acinetobacter baumannii* clinical isolates. mSystems 7. doi:10.1128/msystems.00488-21 PMC8805633 35103489

[108] Maure A, Robino E, Van der Henst C. 2023. The intracellular life of *Acinetobacter baumannii**.* Trends Microbiol 31:1238–1250. doi:10.1016/j.tim.2023.06.007 37487768

[109] Distel JS, Di Venanzio G, Mackel JJ, Rosen DA, Feldman MF. 2023. Replicative *Acinetobacter baumannii* strains interfere with phagosomal maturation by modulating the vacuolar pH. PLoS Pathog 19:e1011173. doi:10.1371/journal.ppat.1011173 PMC10286980 37294840

[110] Ren X, Palmer LD. 2023 a. Acinetobacter metabolism in infection and antimicrobial resistance. Infect Immun 91:e0043322. doi:10.1128/iai.00433-22 PMC10269061 37191522

[111] Rossi E, Longo F, Barbagallo M, Peano C, Consolandi C, Pietrelli A, Jaillon S, Garlanda C, Landini P. 2016. Glucose availability enhances lipopolysaccharide production and immunogenicity in the opportunistic pathogen *Acinetobacter baumannii*. Future Microbiol 11:335–349. doi:10.2217/fmb.15.153 26934424

[112] Ren X, Palmer LD. 2023. *Acinetobacter* metabolism in infection and antimicrobial resistance. Infect Immun 91:e00433–22. doi:10.1128/iai.00433-22 PMC10269061 37191522

[113] Debruyne C, Hodge L, Hokamp K, Ershova AS, Kröger C, Salcedo SP. 2026. Differential roles of the type I and II secretion systems for the intracellular ABC141 *Acinetobacter baumannii* infection, which elicits an atypical hypoxia response in endothelial cells. PLoS Pathog 22:e1013265. doi:10.1371/journal.ppat.1013265 PMC12912691 41662432

[114] Hazen JE, Di Venanzio G, Hultgren SJ, Feldman MF. 2023. Catheterization of mice triggers resurgent urinary tract infection seeded by a bladder reservoir of *Acinetobacter baumannii*. Sci Transl Med 15:eabn8134. doi:10.1126/scitranslmed.abn8134 PMC10464790 36630484

[115] Di Venanzio G, Flores-Mireles AL, Calix JJ, Haurat MF, Scott NE, Palmer LD, Potter RF, Hibbing ME, Friedman L, Wang B, Dantas G, Skaar EP, Hultgren SJ, Feldman MF. 2019. Urinary tract colonization is enhanced by a plasmid that regulates uropathogenic *Acinetobacter baumannii* chromosomal genes. Nat Commun 10:2763. doi:10.1038/s41467-019-10706-y PMC6591400 31235751

[116] Breslow JM, Meissler JJ, Hartzell RR, Spence PB, Truant A, Gaughan J, Eisenstein TK. 2011. Innate immune responses to systemic *Acinetobacter baumannii* infection in mice: neutrophils, but not interleukin-17, mediate host resistance. Infect Immun 79:3317–3327. doi:10.1128/IAI.00069-11 PMC3147579 21576323

[117] van Faassen H, KuoLee R, Harris G, Zhao X, Conlan JW, Chen W. 2007. Neutrophils play an important role in host resistance to respiratory infection with *Acinetobacter baumannii* in mice. Infect Immun 75:5597–5608. doi:10.1128/IAI.00762-07 PMC2168347 17908807

[118] Fleming ID, Krezalek MA, Belogortseva N, Zaborin A, Defazio J, Chandrasekar L, Actis LA, Zaborina O, Alverdy JC. 2017. Modeling *Acinetobacter baumannii* wound infections: the critical role of iron. J Trauma Acute Care Surg 82:557–565. doi:10.1097/TA.0000000000001338 PMC5322184 28030490

[119] Jackson-Litteken CD, Di Venanzio G, Janet-Maitre M, Castro ÍA, Mackel JJ, Wilson LD, Rosen DA, López CB, Feldman MF. 2025. A chronic *Acinetobacter baumannii* pneumonia model to study long-term virulence factors, antibiotic treatments, and polymicrobial infections. Nat Commun 16:7617. doi:10.1038/s41467-025-62655-4 PMC12356904 40817088

[120] Karakonstantis S, Kritsotakis EI. 2021. Systematic review and meta-analysis of the proportion and associated mortality of polymicrobial (vs monomicrobial) pulmonary and bloodstream infections by *Acinetobacter baumannii* complex. Infection 49:1149–1161. doi:10.1007/s15010-021-01663-0 34260054

[121] Timsit JF, Garrait V, Misset B, Goldstein FW, Renaud B, Carlet J. 1993. The digestive tract is a major site for *Acinetobacter baumannii* colonization in intensive care unit patients. J Infect Dis 168:1336–1337. doi:10.1093/infdis/168.5.1336 8228382

[122] Harris AD, Johnson JK, Pineles L, O’Hara LM, Bonomo RA, Thom KA. 2019. Patient-to-patient transmission of *Acinetobacter baumannii* gastrointestinal colonization in the intensive care unit. Antimicrob Agents Chemother 63:00392–19. doi:10.1128/AAC.00392-19 PMC6658781 31085518

[123] Corbella X, Pujol M, Ayats J, Sendra M, Ardanuy C, Domínguez MA, Liñares J, Ariza J, Gudiol F. 1996. Relevance of digestive tract colonization in the epidemiology of nosocomial infections due to multiresistant *Acinetobacter baumannii**.* Clin Infect Dis 23:329–334. doi:10.1093/clinids/23.2.329 8842272

[124] Blanco N, Harris AD, Rock C, Johnson JK, Pineles L, Bonomo RA, Srinivasan A, Pettigrew MM, Thom KA. 2018. Risk factors and outcomes associated with multidrug-resistant *Acinetobacter baumannii* upon intensive care unit admission. Antimicrob Agents Chemother 62:01631–17. doi:10.1128/AAC.01631-17 PMC5740370 29133567

[125] Wong S-C, Chen JH-K, Chau P-H, So SY-C, AuYeung CH-Y, Yuen LL-H, Chan VW-M, Lam GK-M, Chiu KH-Y, Ho P-L, Lo JY-C, Yuen K-Y, Cheng VC-C. 2022. Gastrointestinal colonization of carbapenem-resistant *Acinetobacter baumannii*: what is the implication for infection control? Antibiotics (Basel) 11:1297. doi:10.3390/antibiotics11101297 PMC9598245 36289955

[126] Ren X, Clark RM, Bansah DA, Varner EN, Tiffany CR, Jaswal K, Geary JH, Todd OA, Winkelman JD, Friedman ES, Jarrett RN, Zemel BS, Wu GD, Zackular JP, DePas WH, Behnsen J, Palmer LD. 2025. Amino acid competition shapes *Acinetobacter baumannii* gut carriage. Cell Host & Microbe 33:1396–1411. doi:10.1016/j.chom.2025.07.003 PMC12375414 40763731

[127] Aslam S, Courtwright AM, Koval C, Lehman SM, Morales S, Furr C-LL, Rosas F, Brownstein MJ, Fackler JR, Sisson BM, Biswas B, Henry M, Luu T, Bivens BN, Hamilton T, Duplessis C, Logan C, Law N, Yung G, Turowski J, Anesi J, Strathdee SA, Schooley RT. 2019. Early clinical experience of bacteriophage therapy in 3 lung transplant recipients. Am J Transplant 19:2631–2639. doi:10.1111/ajt.15503 PMC6711787 31207123

[128] Wang W-X, Wu J-Z, Zhang B-L, Yu J-Y, Han L-M, Lu X-L, Li H, Fu S-Y, Ren Y-Y, Dong H, Xu Y, Wang G-T, Gao J-H, Wang C, Chen X-Z, Liu D-X, Huang Y, Yu J-H, Wang S-W, Yang Y-F, Chen W. 2024. Phage therapy combats pan drug-resistant *Acinetobacter baumannii* infection safely and efficiently. Int J Antimicrob Agents 64:107220. doi:10.1016/j.ijantimicag.2024.107220 38810939

[129] Koncz M, Stirling T, Hadj Mehdi H, Méhi O, Eszenyi B, Asbóth A, Apjok G, Tóth Á, Orosz L, Vásárhelyi BM, et al.. 2024. Genomic surveillance as a scalable framework for precision phage therapy against antibiotic-resistant pathogens. Cell 187:5901–5918. doi:10.1016/j.cell.2024.09.009 39332413

[130] Essam K, Kamel AG, Zaki BM, Elhadidy M, Aziz AAA, Fayed A, Roshdy T, El-Shibiny A. 2025. Isolation and characterization of phages ΦZC2 and ΦZC3 against carbapenem-resistant *Acinetobacter baumannii*, and efficacy of ΦZC3 on A549 cells. Virol J 22:262. doi:10.1186/s12985-025-02885-6 PMC12308983 40739242

[131] McCalla AJ, Walker FC, Bisaro F, Rodriguez-Anavitate M, Johannesman A, Di Venanzio G, Feldman MF, LeRoux M. 2025. *Acinetobacter* phages use distinct strategies to breach the capsule barrier. PLoS Pathog 21:e1013536. doi:10.1371/journal.ppat.1013536 PMC12507263 41021605

[132] Orozco-Ochoa AK, González-Gómez JP, Quiñones B, Castro-del Campo N, Valdez-Torres JB, Chaidez-Quiroz C. 2025. Bacteriophage Indie resensitizes multidrug-resistant *Acinetobacter baumannii* to antibiotics *in vitro*. Sci Rep 15:11578. doi:10.1038/s41598-025-96669-1 PMC11971354 40185918

[133] Rastegar S, Skurnik M, Tadjrobehkar O, Samareh A, Samare-Najaf M, Lotfian Z, Khajedadian M, Hosseini-Nave H, Sabouri S. 2024. Synergistic effects of bacteriophage cocktail and antibiotics combinations against extensively drug-resistant *Acinetobacter baumannii**.* BMC Infect Dis 24:1208. doi:10.1186/s12879-024-10081-0 PMC11515142 39455951

[134] Gordillo Altamirano FL, Kostoulias X, Subedi D, Korneev D, Peleg AY, Barr JJ. 2022. Phage-antibiotic combination is a superior treatment against *Acinetobacter baumannii* in a preclinical study. EBioMedicine 80:104045. doi:10.1016/j.ebiom.2022.104045 PMC9097682 35537278

[135] Wang Y, Li L, Liang Y, Xu K, Ye Y, He M. 2025. Phage therapy for *Acinetobacter baumannii* infections: a review on advances in classification, applications, and translational roadblocks. Antibiotics (Basel) 14:1134. doi:10.3390/antibiotics14111134 PMC12649759 41301629

[136] Bisaro F, Jackson-Litteken CD, McGuffey JC, Hooppaw AJ, Bodrog S, Jebeli L, Janet-Maitre M, Ortiz-Marquez JC, van Opijnen T, Scott NE, Di Venanzio G, Feldman MF. 2024. Diclofenac sensitizes multi-drug resistant *Acinetobacter baumannii* to colistin. PLoS Pathog 20:e1012705. doi:10.1371/journal.ppat.1012705 PMC11620633 39571043

[137] Selvam PK, Mohan S, Vasudevan K. 2026. Synergistic interaction of diclofenac with colistin enhances antimicrobial efficacy against multidrug-resistant *Acinetobacter baumannii**.* The Microbe 11:100706. doi:10.1016/j.microb.2026.100706

[138] Fu Q, Liu S, Hu P, Chen H, Zheng J, Shi S, Xu M, Zhou T, Sun Y. 2024. Diclofenac sodium restores the sensitivity of colistin-resistant gram-negative bacteria to colistin. ACS Infect Dis 10:2860–2869. doi:10.1021/acsinfecdis.4c00207 39073350

[139] Ni W, Shao X, Di X, Cui J, Wang R, Liu Y. 2015. *In vitro* synergy of polymyxins with other antibiotics for *Acinetobacter baumannii*: a systematic review and meta-analysis. Int J Antimicrob Agents 45:8–18. doi:10.1016/j.ijantimicag.2014.10.002 25465524

[140] Gezmu AA, Tesfaye AW, Coates ARM. 2026. The role of combination antibiotic therapy in combatting drug-resistant *Acinetobacter baumannii* infections: a systematic review of randomised control trials. Antibiotics (Basel) 15:356. doi:10.3390/antibiotics15040356 PMC13112964 42041319

[141] Luo G, Lin L, Ibrahim AS, Baquir B, Pantapalangkoor P, Bonomo RA, Doi Y, Adams MD, Russo TA, Spellberg B. 2012. Active and passive immunization protects against lethal, extreme drug resistant-*Acinetobacter baumannii* infection. PLoS One 7:e29446. doi:10.1371/journal.pone.0029446 PMC3254619 22253723

[142] Jeffreys S, Chambers JP, Yu J-J, Hung C-Y, Forsthuber T, Arulanandam BP. 2022. Insights into *Acinetobacter baumannii* protective immunity. Front Immunol 13:1070424. doi:10.3389/fimmu.2022.1070424 PMC9716351 36466845

[143] Ranjbar A, Rasooli I, Jahangiri A, Ramezanalizadeh F. 2022. Specific egg yolk antibody raised to biofilm associated protein (Bap) is protective against murine pneumonia caused by *Acinetobacter baumannii*. Sci Rep 12:12576. doi:10.1038/s41598-022-16894-w PMC9307575 35869264

